# Familial Primary Biliary Cholangitis With Different Clinical Courses Based on Anti-gp210 Antibody Positivity: A Report of Two Cases

**DOI:** 10.7759/cureus.34275

**Published:** 2023-01-27

**Authors:** Takahiro Nagata, Yasuaki Takeyama, Satoshi Shakado, Fumihito Hirai, Satoshi Nimura

**Affiliations:** 1 Gastroenterology and Medicine, Fukuoka University Faculty of Medicine, Fukuoka, JPN; 2 Pathology, Fukuoka University Chikushi Hospital, Fukuoka, JPN

**Keywords:** first-degree relatives, clinical course, anti-glycoprotein 210 antibody, familial onset, primary biliary cholangitis

## Abstract

A 30-year-old woman (daughter) was diagnosed to have primary biliary cholangitis (PBC) with autoimmune hepatitis (AIH) overlap syndrome. Although she was started on prednisolone and ursodeoxycholic acid (UA), she eventually died of hepatic failure with gastrointestinal hemorrhage seven months after the initial hospitalization. A 60-year-old woman (mother) was diagnosed with PBC with alcoholic liver cirrhosis, was treated with UA, and had no disease progression. These familial PBC patients had different clinical courses. While the mother was negative for the anti-glycoprotein 210 (anti-gp210) antibody, the daughter was positive for the same. These findings suggest that anti-gp210 antibody positivity affects the prognosis of PBC, even in familial cases.

## Introduction

Primary biliary cholangitis (PBC; formerly known as primary biliary cirrhosis [1]) is a rare autoimmune-mediated chronic bile stagnation liver disease that mainly occurs in middle-aged women [2,3]. It is not generally considered a familial disease, but genetic risk factors have been considered as its causes in recent years [4,5]. Nevertheless, there are no case reports on the prognosis of familial-onset PBC in the literature. Glycoprotein 210 (gp210) is a component of nuclear pores [6]. Anti-gp210 antibody, an autoantibody, has high specificity for PBC [7] and has been detected in some PBC cases with anti-mitochondrial antibody (AMA) negativity [8]. Anti-gp210 antibody has been shown to be useful in predicting the prognosis of PBC cases that have progressed to liver failure [9,10].

In this case report, we present the clinical courses of PBC cases involving a mother and daughter that differed greatly based on the presence or absence of the anti-gp210 antibody.

## Case presentation

Case 1: the daughter

A 30-year-old female presented with liver dysfunction two years ago. However, she did not wish to be examined at that time and left without treatment. Two years later, she was referred to our hospital for general fatigue with jaundice and elevated hepatobiliary enzymes. She had ocular conjunctival yellowing and skin pigmentation. She had no history of alcohol consumption. Relevant family history included hepatitis B virus (HBV)-related liver cirrhosis in her grandmother and occult HBV infection in her mother (HBs antigen and serum HBV DNA were negative and HBc antibody was positive). Her blood biochemical test results are shown in Table 1.

**Table 1:** Laboratory test results of the patients on admission Plt: platelets; PT: prothrombin activation; PT-INR: prothrombin time-international normalized ratio; Alb: albumin; T-Bil: total bilirubin; D-Bil: direct bilirubin; AST: aspartate aminotransferase; ALT: alanine aminotransferase; LDH: lactate dehydrogenase; ALP: alkaline phosphatase; GGT: γ-glutamyl transpeptidase; Ch-E: cholinesterase; M2BPGi: Mac-2-binding protein glycan isomer; ANA: antinuclear antibody; AMA: anti-mitochondrial antibody; IgG: immunoglobulin G; IgM: immunoglobulin M; HLA: human leukocyte antigen

Variables	Case 1: daughter	Case 2: mother	Normal value
Plt (/μL)	73,000	228,000	138,000-309,000
PT (%)	48	59	70-120
PT-INR	1.44	1.33	0.85-1.15
Alb (g/dL)	3.0	2.4	4.0-5.2
T-Bil (mg/dL)	7.0	1.9	0.2-1.2
D-Bil (mg/dL)	4.5	1.1	0-0.3
AST (U/L)	213	44	≤30
ALT (U/L)	103	16	≤30
LDH (U/L)	222	245	124-222
ALP (U/L)	613	482	106-322
GGT (U/L)	119	77	≤50
Ch-E (U/L)	86	73	168-470
M2BPGi (COI)	10.46	12.34	<1.0
Hyaluronic Acid (ng/mL)	367.1	376.2	≤50
ANA (dil)	<40 (-)	<40 (-)	<40
AMA M2 (Index)	147 (+)	338 (+)	<7.0
IgG (mg/dL)	2,074	1,556	870-1,700
IgM (mg/dL)	98	197	46-260
HLA DR	4 (+)/8 (+)	8 (+)/9 (+)	
Anti-glycoprotein 210 antibody (U/mL)	101.7 (+)	4.0 (-)	<5.0
Centromere protein-B (U/mL)	1.5 (-)	0.8 (-)	<7.0

**Figure 1:**
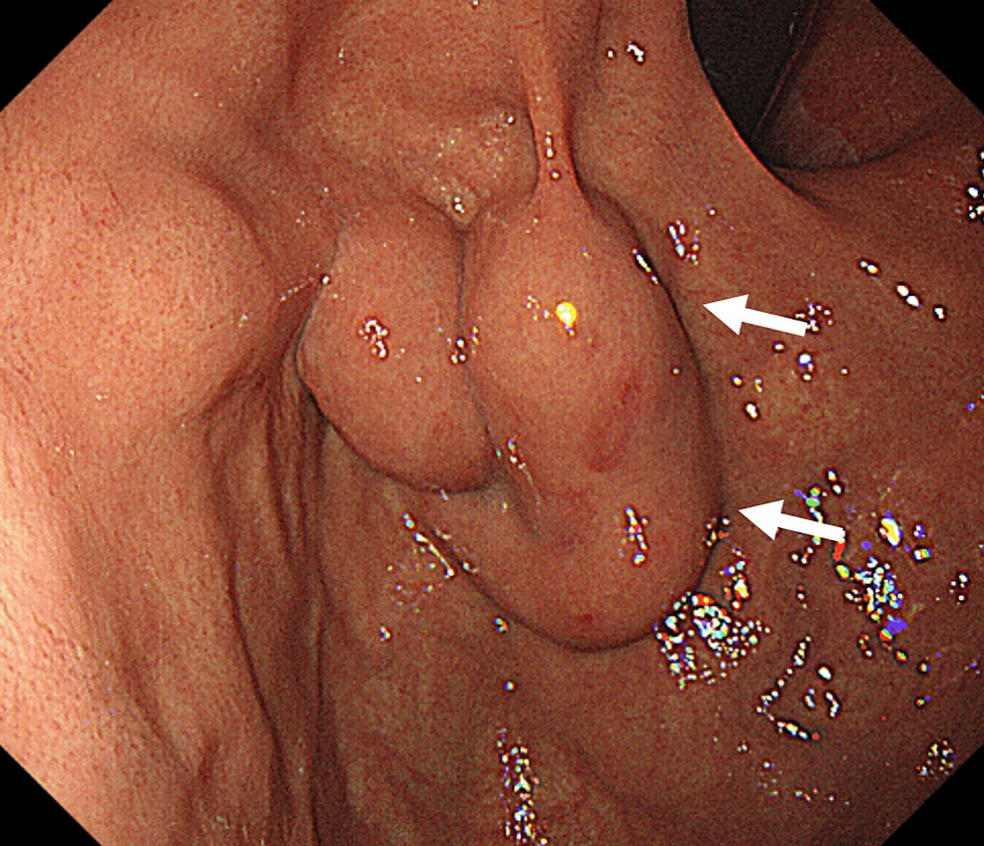
Endoscopic findings of Case 1 The findings show gastric varices with erosions (white arrows) on the surface, but no red color sign is present

All virological tests were negative. CT and MRI revealed liver cirrhosis, while MR elastography showed advanced liver fibrosis. Upper gastrointestinal endoscopy showed gastric varices (Figure 1).

**Figure 2:**
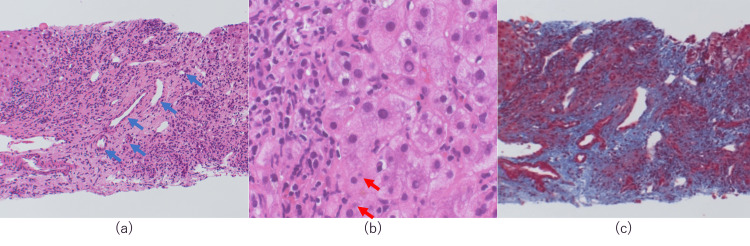
Histological findings of Case 1 (a) Hematoxylin–eosin-stained liver biopsy tissues reveal an increased number of bile ducts (blue arrows) and the presence of chronic non-suppurative destructive cholangitis. (b) Hematoxylin–eosin-stained liver biopsy tissues show the presence of emperipolesis (red arrows) and plasma cell infiltration. (c) Masson-trichrome-stained liver biopsy tissues reveal liver cirrhosis

Histological findings revealed an increased number of bile ducts and the presence of chronic non-suppurative destructive cholangitis (CNSDC) (Figure 2a). Moderate-to-severe lymphocytic and plasma cell infiltration in the portal region, interface hepatitis, hepatocellular rosettes, and emperipolesis were also noted (Figure 2b). Fibrous enlargement of the portal region and fibrous crosslink formation were observed (Figure 2c).

**Figure 3:**
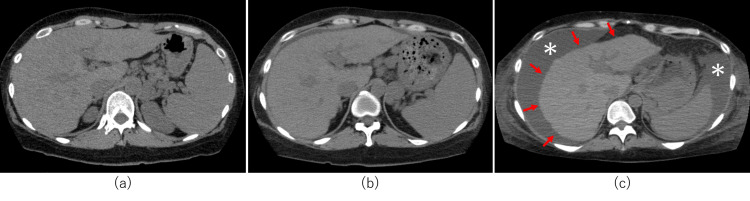
CT images of Case 1 (a) CT image on admission: coarse liver parenchyma, mild splenomegaly, gastro-renal shunt, and mild splenomegaly are seen. (b) CT image after four months: mild hepatic atrophy and slight ascites are present. (c) CT image after eight months: liver atrophy is rapidly progressing (red arrows) and severe ascites is seen (white *) CT: computed tomography

She was graded as Scheuer classification stage 3 [11] and Ludwig classification stage 3 [12]. Nakanuma classification [13] cholangitis activity 2 and hepatitis activity 3 were also observed. The patient was diagnosed to have PBC with autoimmune hepatitis (AIH) overlap syndrome, and started on treatment with 50 mg prednisolone and ursodeoxycholic acid (UA), with tapering of prednisolone. The liver function showed a temporary improvement but then started to worsen after two months, and the patient was registered for liver transplantation and continued to undergo treatment. CT images over time showed rapid liver atrophy and increased ascites (Figure 3).

**Figure 4:**
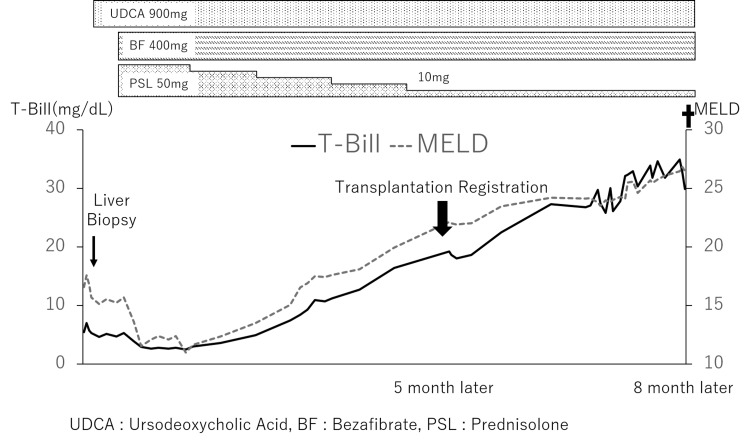
Clinical course of Case 1 The solid line indicates the total bilirubin value, and the dotted line corresponds to the Model for End-Stage Liver Disease score. The thin arrow indicates the date of the liver biopsy and the thick arrow indicates the date of registration for liver transplantation

At seven months after the initial hospitalization, encephalopathy was observed, and the patient had a Model for End-Stage Liver Disease (MELD) score of 25 and a Child-Pugh score (CPS) of 14. The patient subsequently died of advanced liver failure with gastrointestinal bleeding (Figure 4).

Case 2: the mother

**Figure 5:**
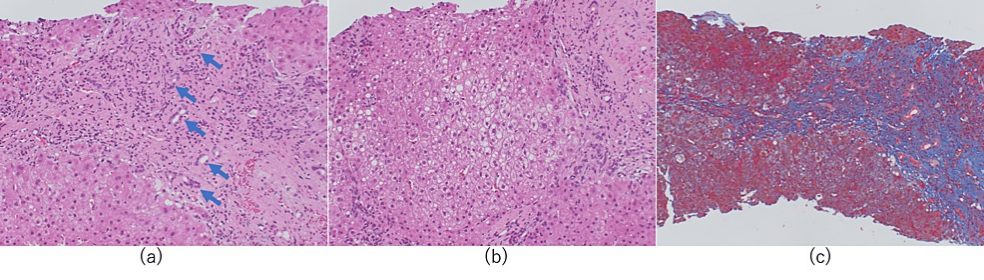
Histological findings of Case 2 (a) Hematoxylin–eosin-stained liver biopsy tissues reveal infiltration of lymphocytes and chronic non-suppurative destructive cholangitis (blue arrows) in the portal region. (b) Hematoxylin–eosin-stained liver biopsy tissues show the presence of many Mallory bodies in the hepatic lobules, indicating the influence of alcoholic hepatitis. (c) Masson-trichrome-stained liver biopsy tissues reveal liver cirrhosis

A 60-year-old female presented with alcoholic liver cirrhosis two years previously. She consulted her original doctor for anemia. Subsequently, she was diagnosed with hepatic encephalopathy and referred to our hospital. She had a Glasgow Coma Scale of III-200 at the time of admission. She also had eyelid conjunctival pallor and ocular conjunctival yellowing. Relevant family history included HBV-related liver cirrhosis in her mother. Her blood biochemical test results are shown in Table 1. Gastrointestinal endoscopy revealed esophageal varices without a red-color sign and bleeding due to gastric antral vascular ectasia. The bleeding was stopped by argon plasma coagulation at the same site. CT and MRI showed a liver cirrhosis pattern. Liver hardness was equivalent to F4, and there was edematous wall thickening in the ascending colon, suggesting portal hypertension. After the stabilization of her condition, a liver biopsy was performed. Histological findings revealed infiltration of lymphocytes and CNSDC in the portal region (Figure 5a). There were also many Mallory bodies in the hepatic lobules, indicating the influence of alcoholic hepatitis with liver cirrhosis (Figures 5b, 5c).

**Figure 6:**
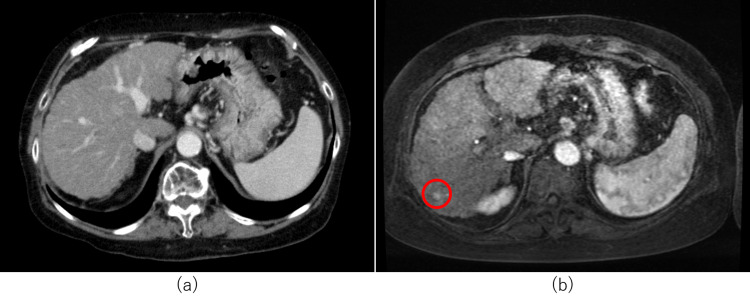
CT and MRI findings of Case 2 (a) Contrast-enhanced CT image at admission: a liver cirrhosis pattern is seen. (b) MRI image with gadolinium ethoxybenzyl diethylenetriaminepentaacetic acid after nine months: there is no progression of hepatic atrophy, but a high/low-pattern hepatocellular carcinoma is seen in liver segment 6 (red circle). Liver hardness is equivalent to F4 CT: computed tomography; MRI: magnetic resonance imaging

She was diagnosed to have alcoholic liver cirrhosis with PBC. After discharge from the hospital, there were no significant changes in her blood test data and CPS. Enhanced MRI was performed at nine months during outpatient follow-up and revealed hepatocellular carcinoma (HCC) in liver segment 6 (Figure 6).

The HCC was treated with microwave ablation. Since then, the patient has been progressing without any significant changes.

## Discussion

PBC is an autoimmune liver disease characterized by AMA positivity and is estimated to occur in 600 people per million population in Japan [14]. UA is considered an effective treatment, and the prognosis of asymptomatic PBC cases does not differ from that in the general population [14]. Intrafamilial PBC occurrence is uncommon, with about 5% of cases reported to be of familial origin [15]. The current familial PBC cases involved a mother and a daughter. Despite treatment with UA in both cases, the daughter showed rapid disease progression, while the mother had a good clinical course.

Anti-gp210 and anti-centromere antibodies are considered to be prognostic predictors for PBC. Anti-gp210 antibody is positive in 20-30% of PBC cases, and the prognosis of these positive cases is poor [16]. Consequently, anti-gp210 antibody positivity can be useful for predicting the prognosis of PBC [9,10]. In the present cases, the mother was negative for anti-gp210 antibody and had a good clinical course, while the daughter was positive for anti-gp210 antibody and had a poor clinical course. Although the daughter had AIH, we further suggest that anti-gp210 antibody positivity is a useful parameter in predicting the prognosis of PBC, even in cases involving first-degree relatives with PBC.

A recently published report has described the clinical characteristics of patients with anti-gp210 antibody-positive PBC [17]. In our cases, the main laboratory test results are shown in Table 1, and the daughter, who was anti-gp210 antibody-positive, had high hepatobiliary enzyme levels and slightly higher IgG. However, this report pertains to two specific cases, and the findings of ANA negativity and AMA being higher in mothers compared to daughters are not consistent with other known studies in the literature [17]. In addition, the possibility that the AIH complication contributed to the difference in the course between the mother and daughter cannot be ruled out, since the pathological examination indicated an AIH complication. Moreover, anti-gp210 antibody-positive cases in AIH-associated PBC have been noted to be resistant to treatment with corticosteroids [18]. However, after hospitalization, there were improvements in clinical symptoms and laboratory data in both cases for a time, and no reliable data could be found to convince us of a rapid exacerbation.

Anti-centromere antibodies are positive in about 20-30% of PBC cases, and positive cases were reported to have higher risks of varices [19,20] and carcinogenesis [14]. Although the mother and daughter in this report were both negative for anti-centromere antibodies, they both had varices, and the mother developed HCC. As for the potential reasons for these findings, the daughter was in the late stage of cirrhosis and the mother had been affected by past alcohol consumption and past HBV infection. Further investigations on the potential risk of PBC cases with positive anti-centromere antibodies are required to gain more insight into the topic.

## Conclusions

We reported two cases of PBC with intrafamilial onset but with very different courses. This report is the first to show that disease progression can vary widely, even between first-degree relatives, and that anti-gp210 antibodies may be useful in predicting the prognosis in these patients. Even cases of familial-onset PBC require careful follow-up, and the evaluation of anti-gp210 antibodies is useful for predicting the prognosis.
